# Pluripotent stem cell-induced skeletal muscle progenitor cells with givinostat promote myoangiogenesis and restore dystrophin in injured Duchenne dystrophic muscle

**DOI:** 10.1186/s13287-021-02174-3

**Published:** 2021-02-12

**Authors:** Wanling Xuan, Mahmood Khan, Muhammad Ashraf

**Affiliations:** 1 Vascular Biology Center, Medical College of Georgia at Augusta University, 1460 Laney Walker Blvd., CB-3712, Augusta, GA 30912 USA; 2 Department of Medicine, Medical College of Georgia at Augusta University, 1460 Laney Walker Blvd, CB-3712, Augusta, GA 30912 USA; 3 Department of Emergency Medicine, Wexner Medical Center, The Ohio State University, Columbus, OH USA

**Keywords:** Duchenne muscular dystrophy, Human induced pluripotent stem cells, Muscle progenitor cells, Histone deacetylase inhibitor, Givinostat, Angiogenesis

## Abstract

**Background:**

Duchenne muscular dystrophy (DMD) is caused by mutations of the gene that encodes the protein dystrophin. A loss of dystrophin leads to severe and progressive muscle wasting in both skeletal and heart muscles. Human induced pluripotent stem cells (hiPSCs) and their derivatives offer important opportunities to treat a number of diseases. Here, we investigated whether givinostat (Givi), a histone deacetylase inhibitor, with muscle differentiation properties could reprogram hiPSCs into muscle progenitor cells (MPC) for DMD treatment.

**Methods:**

MPC were generated from hiPSCs by treatment with CHIR99021 and givinostat called Givi-MPC or with CHIR99021 and fibroblast growth factor as control-MPC. The proliferation and migration capacity were investigated by CCK-8, colony, and migration assays. Engraftment, pathological changes, and restoration of dystrophin were evaluated by in vivo transplantation of MPC. Conditioned medium from cultured MPC was collected and analyzed for extracellular vesicles (EVs).

**Results:**

Givi-MPC exhibited superior proliferation and migration capacity compared to control-MPC. Givi-MPC produced less reactive oxygen species (ROS) after oxidative stress and insignificant expression of IL6 after TNF-α stimulation. Upon transplantation in cardiotoxin (CTX)-injured hind limb of Mdx/SCID mice, the Givi-MPC showed robust engraftment and restored dystrophin in the treated muscle than in those treated with control-MPC or human myoblasts. Givi-MPC significantly limited infiltration of inflammatory cells and reduced muscle necrosis and fibrosis. Additionally, Givi-MPC seeded the stem cell pool in the treated muscle. Moreover, EVs released from Givi-MPC were enriched in several miRNAs related to myoangiogenesis including miR-181a, miR-17, miR-210 and miR-107, and miR-19b compared with EVs from human myoblasts.

**Conclusions:**

It is concluded that hiPSCs reprogrammed into MPC by givinostat possessing anti-oxidative, anti-inflammatory, and muscle gene-promoting properties effectively repaired injured muscle and restored dystrophin in the injured muscle.

**Supplementary Information:**

The online version contains supplementary material available at 10.1186/s13287-021-02174-3.

## Background

Duchenne muscular dystrophy (DMD) is caused by mutations of the gene that encodes the protein dystrophin. Loss of dystrophin leads to severe and progressive muscle wasting in both skeletal and heart muscles. Cell replacement gives a promising hope for muscle atrophy therapy. Satellite cells (SCs) are endogenous skeletal muscle stem cells, which are responsible for muscle maintenance and muscle regeneration after injury [1, 2]. A previous study reported that xenotransplantation of human SCs into mice achieved efficient engraftment and populated the satellite niche [3]. However, a biopsy is needed for the procurement of SCs. In addition, freshly isolated SCs progeny though can be propagated in vitro but their transplantation potential becomes limited during in vitro expansion [4–6]. Therefore, procurement of a larger number of SCs for transplantation becomes an obstacle for clinical application. Human induced pluripotent stem cell (hiPSC)-derived derivatives offer important sources to treat a number of diseases. Efforts have been made in the past few years for the generation of muscle progenitor cells (MPC) from hiPSCs either by genetic modification or small molecules. While the generation of MPC from hiPSCs by viral vectors remains a safety concern, however, recent studies show that Pax7-positive MPC can be generated from hiPSCs by small molecules (CHIR99021, LDN19389, and FGF) [7, 8], but their limited engraftment was observed upon in vivo transplantation [9]. Recently it has been reported that MPC can be generated from teratoma with high engraftment efficiency in the muscle dystrophy model [10]. However, human teratoma-derived MPC poses safety concerns for clinical application. Therefore, it seems more appropriate to look for alternate approaches for inducing MPC from hiPSCs with high engraftment and differentiation properties.

Givinostat is a histone deacetylase inhibitor (HDACi) that has been shown to increase muscle regeneration in a mouse model of DMD [11]. Interestingly, most of the beneficial effects of HDACi arise from its ability to redirect fibroadipogenic lineage commitment toward a myogenic fate [12]. Using genome-wide Chip-seq analysis in myoblasts, it was demonstrated that HDACi induced myogenic differentiation program in myoblasts (i.e., Myosin 7, Enolase 3, and Myomesin1) [13]. Therefore, here, we propose that givinostat could reprogram hiPSCs into MPC which can repair and restore muscle defects.

## Methods

### Human iPSC culture

The Human iPSC cell lines, CYS0105 and DYS0100, from ATCC Company were used. CYS0105 was reprogrammed from human cardiac fibroblasts of a 72-year-old healthy donor (CF-iPSC), while DYS0100 was reprogrammed from human foreskin fibroblasts of a normal newborn (DF-iPSC-1). The third iPS cell line (DF-iPSC-2) was reprogrammed from human dermal fibroblasts (CC-2511, Lonza) of a 45-year-old healthy donor in our lab using Cyto TuneTM iPS 2.0 Sendai Reprogramming Kit (A16517, Thermo Fisher Scientific) as previously described [14]. iPSCs were grown and maintained on vitronectin coated six-well plate in mTeSR1 medium (Stem Cell Technologies) with daily change.

### Differentiation protocols for muscle progenitor cells and their characterization

**Fig. 1:**
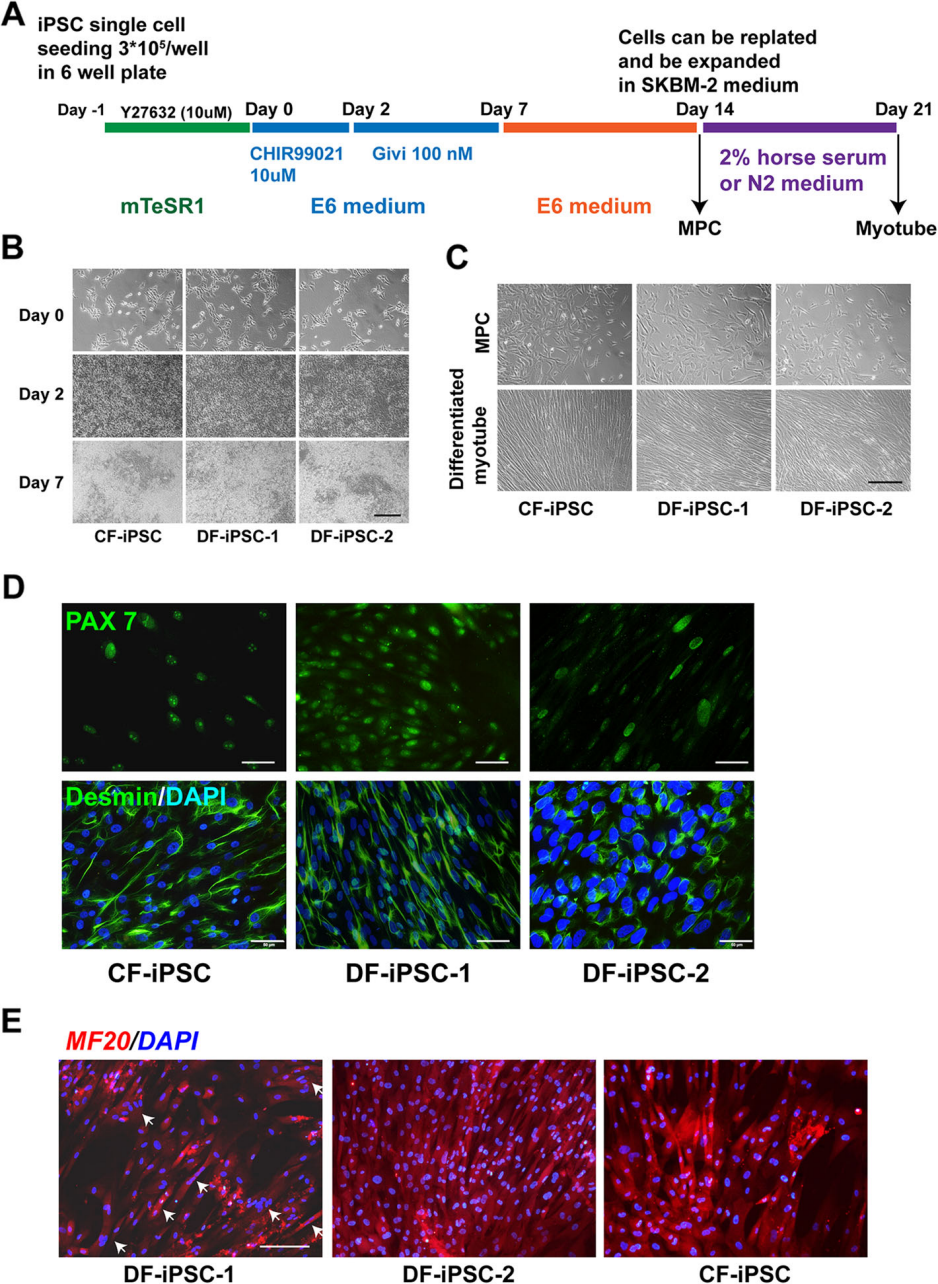
Generation of muscle progenitor cells (MPC) from human iPSC using small molecules. **a** Schematic outline of the generation of MPC from human iPSC using a combination of CHIR99021 and givinostat or CHIR99021 only. **b** Morphology of differentiating cells from 3 human iPSC lines (CF-iPSC, DF-iPSC-1, and DF-iPSC-2) at 7 days. Bar = 200 μm. **c** Morphology of replated MPC and differentiated MPC in muscle differentiation medium from 4 human iPSC lines at day 14. Bar = 200 μm. Characterization of givinostat-induced MPC: **d** The treated hiPSC at day14 expressed Pax7 and desmin. Bar = 100 μm. **e** The differentiated myotubes expressed MF20 as shown by immunostaining. Bar = 100 μm. The white arrows point at the multinuclear cells in fused cells

Human iPSCs at passages 20–30 were used for conversion to MPC. Human iPSCs were dissociated into single cells using Accutase (Stem Cell Technologies) at 37C for 10 min and then were seeded onto a vitronectin-coated six-well plate at 3 × 10^5^ cell/well in mTeSR1 supplemented with 5 μM ROCK inhibitor (Y-27632, Stem Cell Technology) for 24 h. Afterwards, cells were switched to E6 medium (Thermo Fisher Scientific) supplemented with CHIR99021 (10 μM), a glycogen synthase kinase 3 inhibitor, to augment mesoderm induction for 2 days followed by givinostat (100 nM) for 5 days. The differentiating cells were cultured in an E6 medium for 7 days. The schematic outline is shown in Fig. 1a. At day 14, cells were replated on 0.1% galectin-coated coverslips, and expression of Pax7 and desmin were analyzed by immunostaining. MPCs were expanded in SKGM-2 medium plus FGF-2 (2.5 ng/ml), and cells at passages 2–4 were used for experiments. Here, we referred to the givinostat-induced MPC as Givi-MPC. For further muscle maturation, cultured cells were replated and switched to high-glucose DMEM medium supplemented with 2% horse serum and 1% ITS (Thermo Fisher Scientific) or N2 medium for 7 days. Immunostaining of cultured cells for MF20 was performed. To generate control-MPC, a previously reported method using only CHIR99021 and FGF were used [7]. The schematic outline is shown in supplemental Fig. S1. control-MPC were expanded under the same conditions as Givi-MPC, and cells at passages 2–4 were used for experiments. control-MPC and Givi-MPC differentiated from the same human iPS cell line, DYS0100, were used to characterize their proliferation and cell migration properties.

### CCK-8 assay for proliferation

The CCK-8 assay was used for the evaluation of cell proliferation. Briefly, 2000 cells were seeded into 96-well plate per well, and cell proliferation was analyzed at 0 h, 24 h, 48 h, and 72 h by using the CCK-8 kit (ab228554, Abcam) according to the manufacturer’s protocol.

### Colony formation

Thirty cells (single cell) were seeded in one well of six-well plate. After 7 days, cells were stained with crystal violet dye. The number of colonies and size of cell growth were analyzed and compared between the control-MPC and Givi-MPC groups.

### Cell migration

For cell migration experiment, human myoblasts, control-MPC, and Givi-MPC were seeded in a 35-mm dish with culture-insert 2 well (ibidi GmbH company) at 1 × 10^5^/ml concentration in SKGM-2 medium with 2% fetal bovine serum (FBS). The next day, a confluent layer was observed and culture-inserts were removed, and after 24 h, the number of migrated cells was analyzed.

### Human endothelial cell and human myoblast culture

Human aortic endothelial cells (HAEC, CC-2535) and human skeletal myoblasts (HSMM-Muscle Myoblasts, CC-2580) were obtained from Lonza Company. HAECs were maintained in endothelial cell growth medium V-2 (213-500, CELL APPLICATIONS, Inc.), and cells at passages 2–6 were used for experiments. Human myoblasts were maintained in SKGM-2 medium (Lonza), and cells at passages 2–4 were used for experiments.

### Cardiotoxin injury and cell transplantation

Animal experiments were carried out according to the experimental protocol approved by the Augusta University Animal Care and Use Committee. Six to 8-week-old Mdx/SCID mice (Stock No: 018018, The Jackson Laboratory) were used in the present study. One day prior to cell transplantation, mice were anesthetized using 2% isoflurane, and the tibialis anterior (TA) muscle was injured with 50 μl of 10 μM cardiotoxin (Naja mossambica-mossambica, Sigma). The same human iPS cell line, DYS0100, was used to derive control-MPC and Givi-MPC for transplantation. Primary myoblasts, control-MPC, and Givi-MPC were dissociated using Accutase (Stem Cell Technologies) and resuspended in Dulbecco’s phosphate-buffered saline (DPBS) at 1 × 10^5^ per 20 μl. Cells were injected into the left tibialis anterior (TA) muscle while the same volume of DPBS was injected into the right TA as control. In some cases, cells were transfected with green fluorescent protein (GFP) lentivirus (abm company, Canada) for cell tracking. Some Mdx/SCID mice transplanted with Givi-MPC were subjected to CTX reinjury at 2 M after first injury and cell transplantation.

### Immunofluorescence staining for cells

Cells were fixed with 4% PFA, and blocked with 10% FBS, followed by incubation with anti-Pax7 antibody (ab187339, Abcam, 1:300), anti-desmin antibody (ab32362, Abcam, 1:500), and anti-myosin heavy chain antibody (MF20) antibody (Novus, MAB4470, 1:200) respectively at 4 °C overnight and secondary antibody conjugated to Alexa Fluor 594 or Alexa Fluor 488 (Life Technologies) at room temperature for 1 h. Images were taken by a fluorescent microscope (Olympus, Japan).

### Immunofluorescence staining for muscle tissue

After 7 days or 30 days of cell transplantation, Mdx/SCID mice were euthanized and the TA muscles were harvested and fixed with 4% paraformaldehyde (PFA) for 1 h at room temperature and then immersed in 30% sucrose overnight at 4 °C. At day 2, the TA muscles were cryopreserved in an optical cutting temperature (OCT) compound (Tissue Tek) at − 80 °C. TA muscle samples were sliced into 5-μm-thick frozen cross-sections using a Leica CM3050 cryostat. The muscle sections were incubated with primary antibodies including Laminin (L9393, Sigma, 1:500 and L0663, Sigma, 1:300), dystrophin (D8168, Sigma, 1:200), human-specific dystrophin (NBP2-79783, Novus, 1:200), green fluorescent protein (GFP, #2956, Cell Signal Technologies, 1:500), dystrophin (ab15277, Abcam, 1:200), human nuclear antigen (NBP2-34342, Novus, 1:100), CD68 (NB600-985, Novus, 1:200), CD31 (NB600-562, Novus, 1:200), Pax7 (ab187339, Abcam, 1:300), and human LaminA/C (NBP2-59933, Novus, 1:200) at 4 °C overnight respectively and anti-rabbit/mouse/rat secondary antibodies conjugated to Alexa Fluor 594 or Alexa Fluor 647 or Alexa Fluor 488 (Life Technologies) at room temperature for 1 h. Images were taken using a confocal microscope (FV1000, Olympus, Japan). For cell engraftment quantification, 4 sections at 150-μm interval in each TA muscle were analyzed. Dystrophin or laminin staining was used to define the physical boundaries of muscle fibers. The number of muscle fibers and cross-section area was measured using ImageJ with the colocalization plugin (NIH). Capillary density was assessed in 4 sections cut at 150-μm interval by counting CD31-positive vascular structures using a fluorescence microscope at a magnification of × 400. The number of capillaries in each TA muscle was expressed as the number of capillary per field (0.2 mm^2^). For quantification of inflammatory cells, the number of CD68-positive cells was counted in 3 sections cut at 150-μm interval 7 days post-cell transplantation and was expressed as the number of CD68-positive cells per field (0.2 mm^2^). Staining of presynaptic marker α-bungarotoxin (α-BTX) was carried out using α-bungarotoxin, Alexa Fluor™ 594 conjugate (Invitrogen) according to the manufacturer’s instruction.

### Histology

Histology of the muscle was performed by Electron Microscopy and Histology Core of Augusta University. After 7 days or 30 days of cell transplantation, the TA muscle was harvested and embedded in paraffin. Five-micrometer-thick sections of the TA muscle were cut and stained with hematoxylin and eosin (H and E), Masson's trichrome, and Sirius red according to the manufacturer’s protocol (Abcam). Images were taken by a vertical microscope (Olympus, Japan). Fibrosis and necrosis were determined using the ImageJ software (NIH) and expressed as the ratio of the total area of the cross-section and normalized with the ratio of the control lateral TA muscle section. Myofiber necrosis was identified with fragmented sarcoplasm [15] and/or increased inflammatory cell infiltration and was measured using non-overlapping tile images of transverse muscle sections.

### Isolation of extracellular vesicles

Extracellular vesicles (EVs) were isolated using the size exclusion column method as we described previously [16]. Briefly, the conditioned medium was collected, and EVs were isolated by centrifugation at 3000 rpm for 30 min to remove cells and debris, followed by filtration through a 0.22-μm filter to remove the remaining debris. Then, the medium was further concentrated using Amicon Ultra-15 100 kDa centrifugal filter units (Millipore). Isolation of EVs in the concentrated medium was carried out through the qEV size exclusion columns (Izon Science). EV fractions were collected and concentrated by Amicon Ultra-4 10 kDa centrifugal filter (Millipore). The purified EVs were stored at − 80 °C and subsequently characterized by particle size and electron microscopy.

### Concentration and particle size measurement with tunable resistive pulse sensing

Particle size and concentration were analyzed using the tunable resistive pulse sensing (TRPS) technique with a qNano instrument (Izon Science) as described in previous studies [16, 17]. Briefly, particles were counted (at least 600 to 1000 events) at 20 mbar pressure. Beads CPC200 (200 nm) were used for calibration. Data were analyzed using the Izon Control Suite software.

### Transmission electron microscopy

Tissue samples were processed for TEM by the Electron Microscopy and Histology Core Laboratory at Augusta University as described previously [16]. Briefly, EVs suspension was fixed with an equal volume of 8% paraformaldehyde to preserve ultrastructure. Ten microliters of suspended/fixed exosomes was applied to a carbon-formvar-coated 200 mesh copper grid and allowed to stand for 30–60 s. The excess was absorbed by Whatman filter paper. Ten microliters of 2% aqueous uranyl acetate was added and treated for 30 s. Grids were allowed to air dry before being examined in a JEM 1230 transmission electron microscope (JEOL USA Inc., Peabody, MA) at 110 kV and imaged with an UltraScan 4000 CCD camera & First Light Digital Camera Controller (Gatan Inc., Pleasanton, CA).

### RNA extraction and PCR array

Total RNA from cells was isolated using miRNeasy Kit (Qiagen). Reverse transcription was performed using QuantiTect Reverse Transcription kit (Qiagen). Human cell motility RT2 profiler PCR Array (Qiagen) for control-MPC and Givi-MPC was performed. Data were analyzed using RT2 Profiler PCR Array Data Analysis Webportal (Qiagen). Genes with a fold change > 2.0 were considered overexpressed.

### RNA extraction from EVs and miRNA array analysis

Total RNA from EV was isolated using the miRNeasy Micro Kit (Qiagen). The miRNA array analysis was performed in the Integrated Genomics and High Performance Computer Server Center at Augusta University. RNA purity and concentration were evaluated by spectrophotometry using Nanodrop ND-1000 (Thermo Fisher Scientific). The quality and size of small RNA were assessed by the Agilent 2100 Bioanalyzer (Agilent Technologies, Santa Clara, CA). One hundred thirty nanograms of total RNA was labeled with biotin using the FlashTag Biotin HSR RNA Labeling Kit (Applied Biosystems) according to the manufacturer’s procedure. The labeled samples were then hybridized to the GeneChip miRNA 4.0 array (Thermo Fisher) that contains 2578 and 2025 human mature and premature miRNA, respectively. Array hybridization, washing, and scanning of the arrays were carried out according to Affymetrix’s recommendations. Data was obtained in the form of CEL file. The CEL files were imported into Partek Genomic Suites version 6.6 (Partek, St. Louis, MO) using a standard import tool with RMA normalization. The differential expressions were calculated using ANOVA of the Partek Package.

### Tube formation assay

Human aortic endothelial cells (HAEC, 1 × 10^5^ cells/well) were seeded on Matrigel (Corning) in a 24-well plate and treated with or without 1 μg EVs from Givi-MPC, control-MPC, or human myoblast in EGM-2 V basal medium (Lonza). After 16 h, cells in Matrigel were stained with Calcein AM, and images were taken with a fluorescent microscope. Tube formation was analyzed by the ImageJ software with the angiogenesis analyzer plugin (NIH).

### Reactive oxygen species and inflammatory cytokine measurement

Oxygen reactive species were measured by using dihydrorhodamine 123 which is uncharged non-fluorescent dye and a derivative of rhodamine 123 (R123). This probe passively enters into the cells, and is oxidized by ROS, to form R123. R123 is a cationic green fluorescent dye that can accumulate and localize into the mitochondria. The change in fluorescence intensity reflects the change in relative levels of ROS production. control-MPC and Givi-MPC were treated with H_2_O_2_ (100 μM) for 24 h. Then, cells were stained with dihydrorhodamine 123 (20 μM) for the measurement of ROS. Fluorescence intensity was analyzed using the ImageJ software. For the measurement of an inflammatory cytokine, IL6, control MPC, and Givi-MPC were treated with 10 ng/ml TNFα for 24 h and processed for Western blotting.

### Western blotting

Five to 10 μg protein was separated by SDS/PAGE and transferred to the PVDF membrane (BioRad). The membranes were incubated with primary antibodies against the following proteins overnight at 4 °C: rabbit-anti-IL-6 (#12153, CST) and rabbit anti-Tubulin (#15115, CST). For histone acylation analysis, the membranes were incubated with primary antibodies: Acetyl-Histone H3 (Lys9) (#9649, CST) antibody, Histone H3 (# 4499, CST) antibody, Acetyl-Histone H4 (Lys8) antibody (#2594, CST), and Histone H4 antibody (#13919, CST). After three times of wash, the membranes were incubated with an anti-rabbit/goat peroxidase-conjugated secondary antibody. Immunoreactive bands were visualized by the enhanced chemiluminescence method (Bio-Rad) with a Western blotting detection system (Fluorchem E, ProteinSimple, USA) and were quantified by densitometry with the ImageJ software.

### Statistical analysis

Data were expressed as mean ± SD. After the test for normality, statistical analysis of differences among the different groups was compared by ANOVA with Bonferroni’s correction for multiple comparisons. The percentage of different sizes of the colony was compared using the chi-squared test. The differences were considered statistically significant at *P* < 0.05. Statistical analyses were performed using Graphpad Prism 6.0 (Chicago, USA).

## Results

### Generation of muscle progenitor cells from human iPSC using small molecules

As outlined in Fig. 1a, we used 3 iPSC lines from healthy donors of different ages, and one iPSC line from a DMD patient with frameshift deletions of exons 3–7 in the dystrophin gene for MPC generation. After treatment with CHIR99021 for 2 days, the morphology of the differentiating cells from all cell lines was dramatically altered suggesting epithelial to mesenchymal transition (EMT) (Fig. 1b). Following treatment with givinostat for 5 days, cells became confluent and clustered (Fig. 1b). Figure 1c showed the morphology of differentiated MPC after replating and terminal muscle differentiation for 7 days in 2% horse serum differentiation medium. The MPC during terminal differentiation were elongated (Fig. 1c). Immunostaining revealed that the MPC derived from all iPSC lines were positive for the myogenic markers Pax7 and desmin (Fig. 1d) and the terminal differentiated cells expressed MF20 and displayed cell fusion (Fig. 1e), indicating their myogenic differentiation potential.

### Givinostat-induced MPC expressed high proliferation and motility properties in vitro

**Fig. 2:**
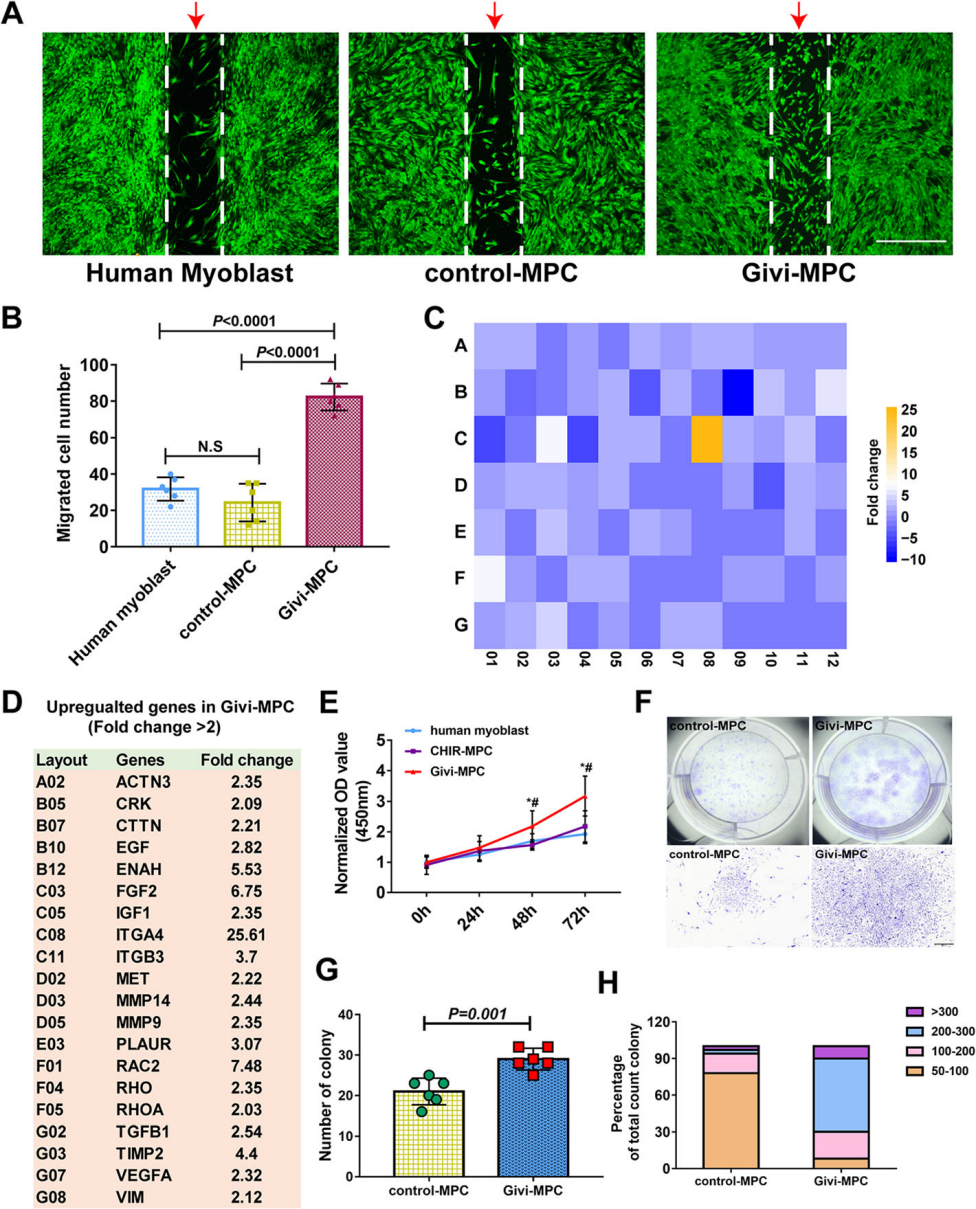
Givi-MPC exhibited superior proliferation and migration capacity. **a** Representative images and **b** quantitative estimate of cell migration by adult human myoblasts and control-MPC, Givi-MPC (arrow). Cells were stained with Calcein AM (green). Bar = 1 mm. **b** Quantitative estimate of migrated cells. Givi MPC showed the highest number of cells migrated as compared with human myoblasts (*P* < 0.0001) or CHIR99021 induced MPC (*P* < 0.0001). No significant difference was observed between human myoblasts and control-MPC. **c** Heat map of the human RT2 motility PCR array. **d** Differentially expressed genes related to migration in Givi-MPC vs. control-MPC using human cell motility PCR array. **e** Proliferation curves of human myoblasts vs. MPC using CCK-8 assay. *P < 0.05; ^#^*P* < 0.05 vs. control-MPC. *n* = 6. **f** Morphology of MPC colonies. Bar = 500 μm. Number of colonies (**g**) and percentage of colonies with different cell number in color (**h**). control-MPC: CHIR99021-induced MPC; Givi-MPC: CHIR99021 and givinostat-induced MPC

We explored whether MPC were proliferative and possessed self-renewal and motility properties. Using migration assay, compared to normal adult human myoblasts or control-MPC, Givi-MPC exhibited superior migration capability in low-serum medium culture (Fig. 2a) with the highest number migrated compared to other MPC (Fig. 2b). Genes related to migration as determined by cell motility PCR array increased multifold in Givi-MPC as compared with other MPC (ITGA4 (25.61-fold), RAC2 (7.48-fold), FGF2 (6.75-fold), and ENAH (5.53-fold). Figure 2c and d show a heatmap which identified a list of upregulated genes related to cell migration (> 2-fold). In addition, the CCK-8 assay at 48 h and 72 h time points Givi-MPC showed higher OD value compared with human myoblasts and control-MPC (Fig. 2e). Colonies formed by Givi-MPC were bigger with higher cell density compared to control MPC (Fig. 2f). Quantitative data (Fig. 2g, h) showed that Givi-MPC formed more colonies containing a higher density of cells (> 200 cells) compared to control MPC. Although the CCK-8 assay was used for cell viability, these data in combination with colony formation assay support the notion that Givi-MPC possessed high proliferation and self-renewal capabilities. In comparison with control MPC, Givi-MPC also possessed anti-oxidative and anti-inflammatory properties which were supported by less ROS production after oxidative stress and insignificant expression of inflammatory cytokine, IL6 after TNF-α stimulation (Fig. S2). Furthermore, givinostat significantly promoted acetylation of histone 3 (H3) at lysine 9, till maturation of induced MPC at day 14 compared with CHIR99021 treatment alone (Fig.S3). We can infer that changes in the gene expression for cell differentiation are at least partly due to the acetylation of H3 which may contribute to the specific properties of Givi-MPC for myoangiogenesis.

### In vivo engrafted Givi-MPC were integrated into injured Mdx muscle and restored dystrophin

**Fig. 3:**
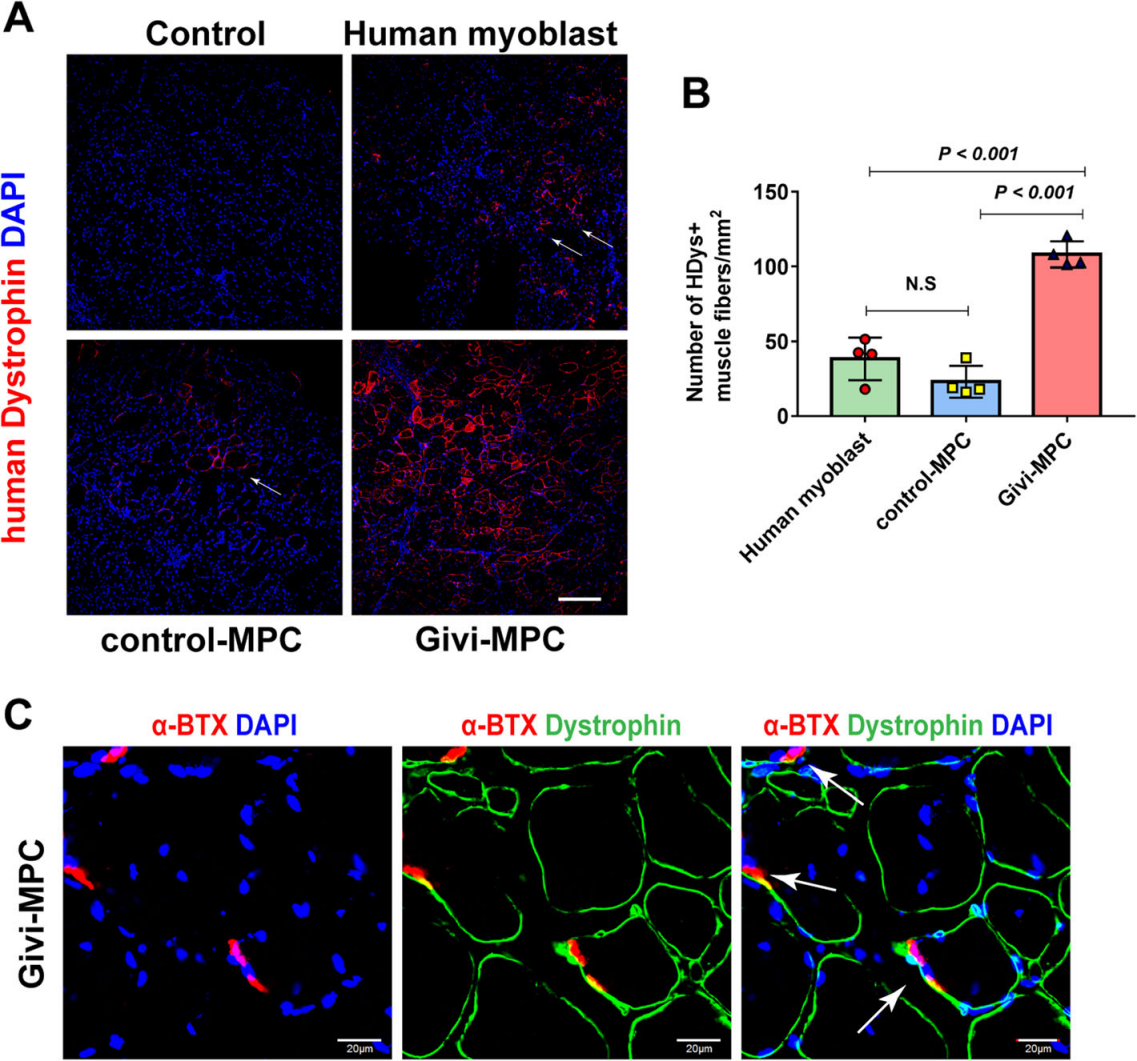
In vivo myogenic potential of different MPC in Mdx/SCID mice with CTX injury. **a** Restoration of dystrophin in Mdx/SCID mice by MPC transplantation at 1 M after CTX injury with human-specific dystrophin staining. Human dystrophin (red), DAPI (blue). Bar = 200 μm. **b** Quantitation of engrafted human dystrophin-positive (HDYs) fibers at 1 M: (*n* = 4). **c** Cross-section showing pre-synaptic staining with α-bungarotoxin in dystrophin-positive fibers (*n* = 3). Bar = 20 μm

Here, we tested the regenerative potential of the donor cells in Mdx/SCID mice following CTX injury. Human myoblasts, control-MPC, or Givi-MPC were transplanted into Mdx/SCID mice with CTX injury. We used human-specific dystrophin antibody to detect muscle regeneration or revertant muscle fibers in the injured muscle of Mdx/SCID mice. One month post-transplant, Givi-MPC showed an immense engraftment capacity and restoration of dystrophin compared with the treatment with control-MPC and human myoblasts as confirmed by human-specific dystrophin staining (Fig. 3a, b). Fig.S4A also showed that the engrafted Givi-MPC (GFP-positive) expressed dystrophin. The Givi-MPC-treated TA muscle had also a significantly high number of dystrophin-positive muscle fibers which were stained with human- or non-human-specific dystrophin antibodies (Fig. 3c, Fig.S4B, and S4C). To determine the functionality of the newly formed muscle fibers from Givi-MPC, we tested whether they were integrated and innervated into the recipient environment. Positive staining of presynaptic marker α-BTX was observed in close proximity to dystrophin-positive muscle fibers in the Givi-MPC-treated TA muscle, suggesting the formation of the neuromuscular junction in these muscle fibers and integration of donor cells in the TA muscle (Fig. 3c).

### Givi-MPC reduced inflammation, muscle necrosis, and fibrosis in Mdx/SCID mice post-CTX injury

**Fig. 4:**
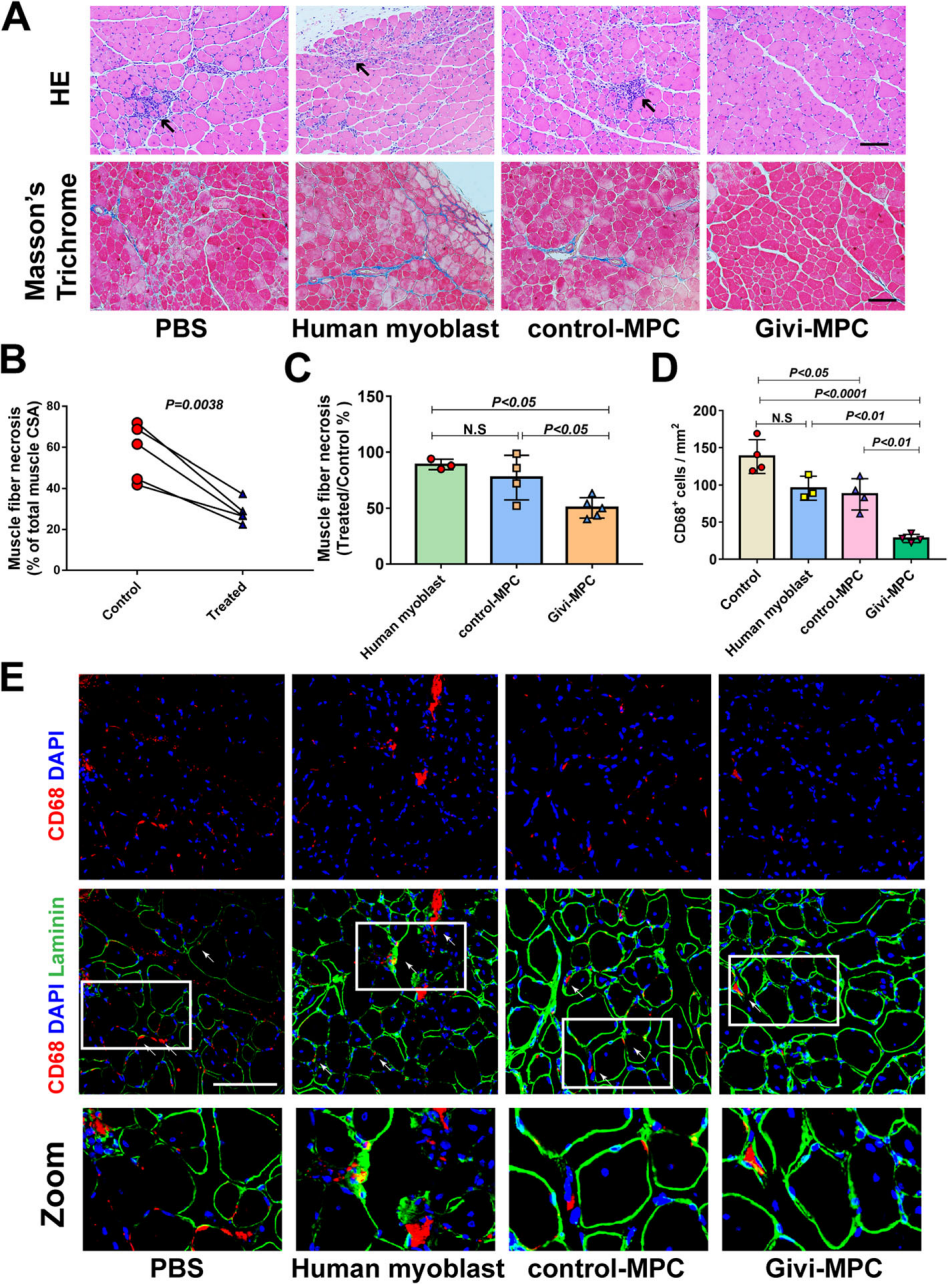
Givi-MPC decreased inflammation and muscle necrosis in Mdx/SCID mice 7 days after CTX injury. **a** Representative images of HE and Masson's trichrome staining in Mdx/SCID mice after human myoblasts or control MPC or Givi-MPC transplantation 7 days after CTX injury. Black arrows point at infiltrated inflammatory cells. **b**, **c** Quantification of TA muscle fiber necrosis after treatment with MPC 7 days after CTX injury. Human myoblasts: *n* = 3; control-MPC: *n* = 4; Givi-MPC: *n* = 5. **d** Quantification of CD68-positive cells in TA muscle following MPC transplantation 7 days after CTX injury. *n* = 4. **e** Representative images of macrophages (red, CD68) and human cells in TA CTX-injured muscle of Mdx/SCID mice following MPC transplantation. The boxed area is shown below at high magnification. Bar = 100 μm

**Fig. 5:**
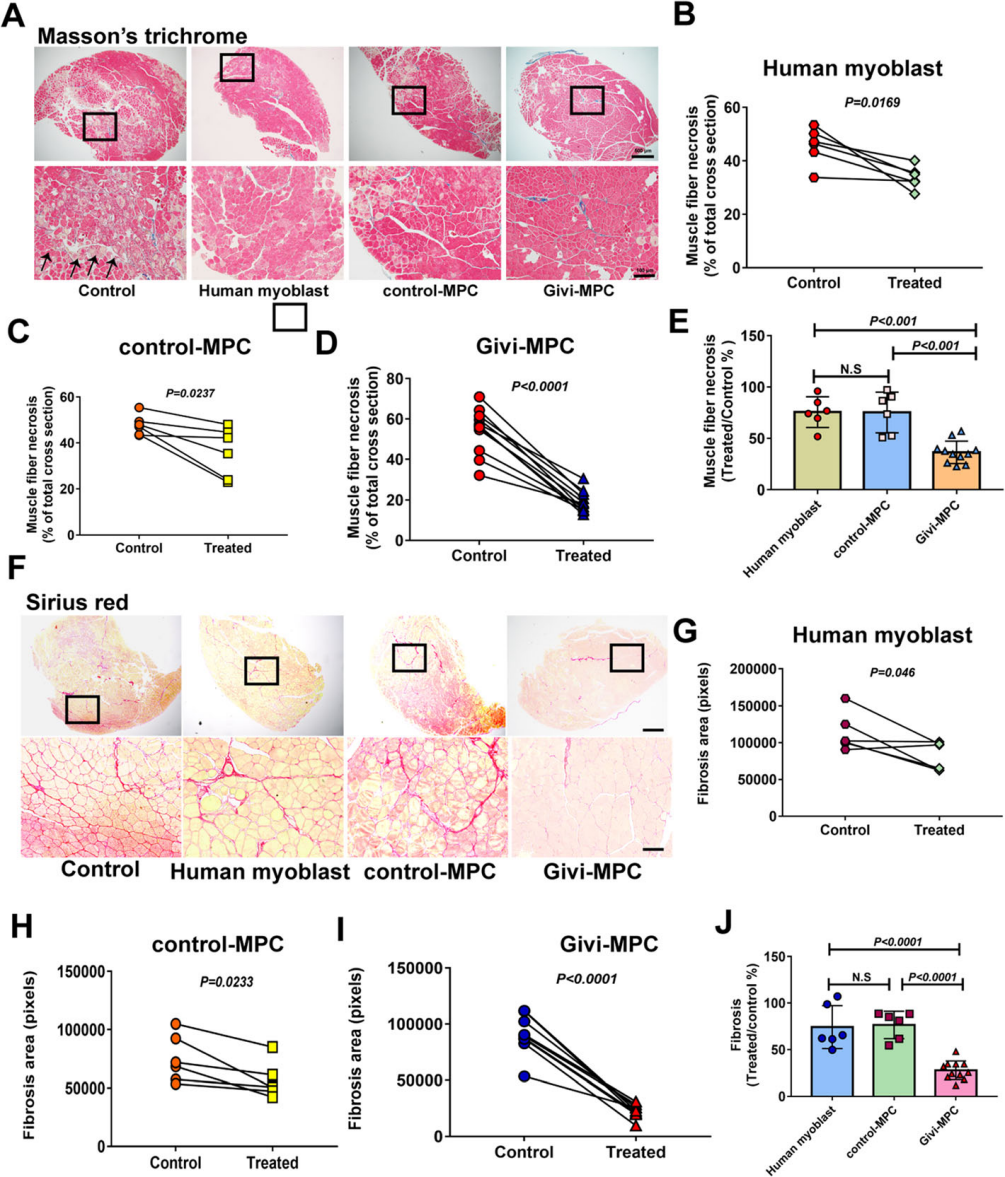
Givi-MPC decreased muscle necrosis and fibrosis in Mdx/SCID mice 1 M after CTX injury. **a** Representative images of HE and Masson's trichrome staining in Mdx/SCID mice after transplantation with human myoblasts or control MPC or Givi-MPC 1 M after CTX injury. Bar = 500 μm (× 4) and bar = 100 μm (× 20). Quantification of necrotic muscle fibers after treatment with human myoblasts (**b**), control MPC (**c**), and Givi-MPC (**d**) 1 M after CTX injury. **e** Comparison of muscle necrosis among human myoblasts or control-MPC- or Givi-MPC-transplanted Mdx/SCID mice. **f** Representative images of tissue stained with Sirius red after transplantation with MPC. Bar = 500 μm (× 4) and bar = 100 μm (× 20). The boxed area is shown below at high magnification. Quantification of muscle fiber fibrosis in TA muscle treated with human myoblasts (**g**), control-MPC (**h**), and Givi-MPC (**i**) 1 M after CTX injury. **j** Muscle fibrosis after transplantation of different MPC. *n* = 6 in the human myoblasts and control MPC groups and *n* = 11 in the Givi-MPC group

**Fig. 6:**
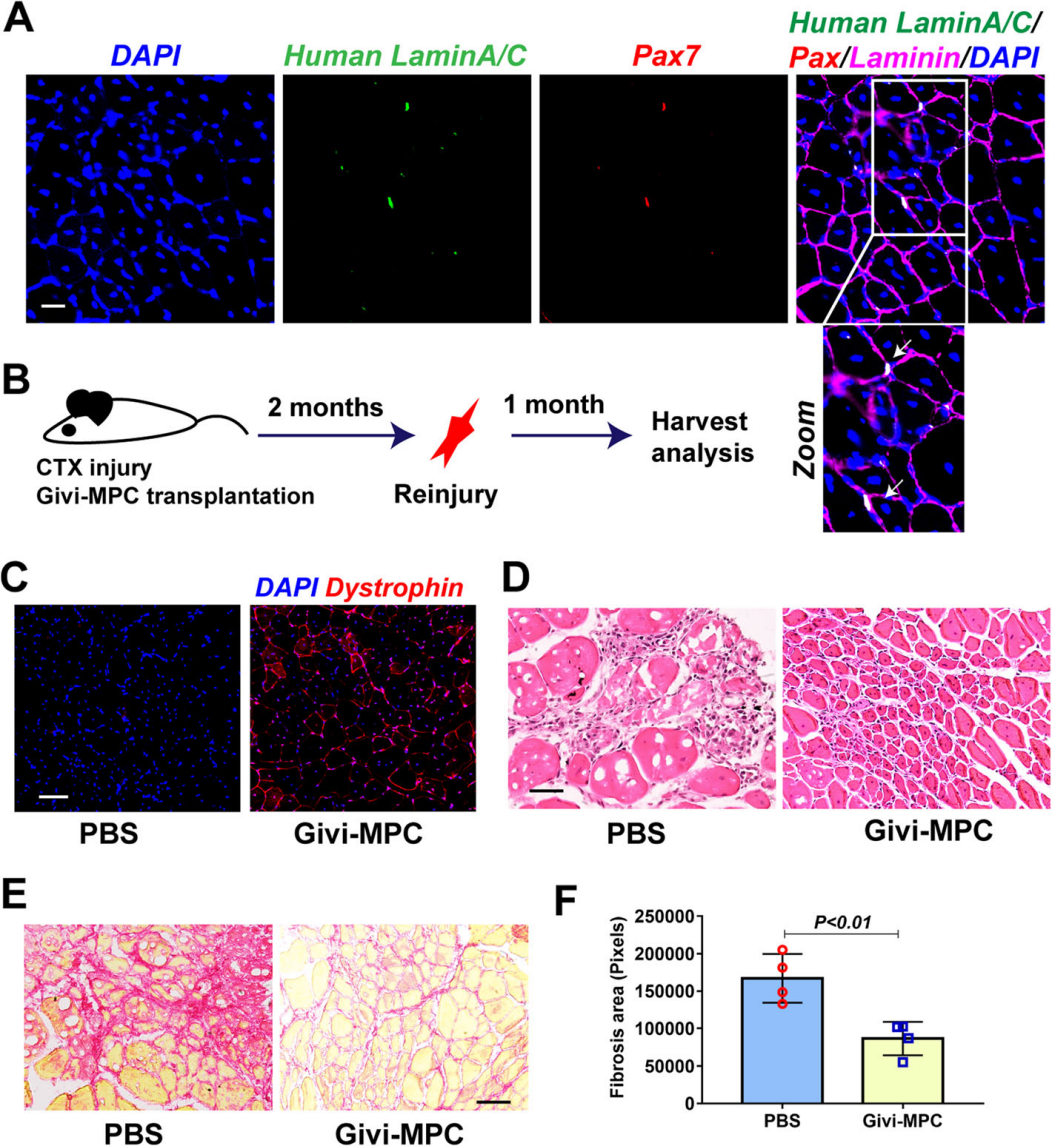
Givi-MPC repopulated the muscle stem cell pool. **a** Muscle cells positive for Pax7 (red) and human laminA/C (green) cell under the basal lamina from Mdx/SCID mice after 1 M post-Givi-MPC transplantation. Bar = 20 μm. The boxed area is zoomed below. **b** Schematic outline of reinjury experiment. **c** 1 M after reinjury, the expression of dystrophin in Givi-MPC-treated TA muscle tissue. Bar = 50 μm. **d** Representative HE-stained images of Givi-MPC-treated TA muscle tissue and contralateral PBS-treated TA muscle tissue. Bar = 50 μm. **e** Representative images of tissue stained with Sirius red after reinjury. Bar = 50 μm. **f** Quantification of muscle fiber fibrosis in TA muscle treated with PBS and Givi-MPC after reinjury. *n* = 4

Hematoxylin and eosin- and Masson's trichrome-stained tissue revealed the infiltration of inflammatory cells and necrotic muscle fibers in Mdx/SCID mice 7 days post-CTX injury (Fig. 4a). A significant decrease in muscle necrosis was observed in the Givi-MPC-treated TA muscle compared to the contralateral PBS-treated TA muscle (Fig. 4b). Among different transplanted MPC, Givi-MPC reduced muscle necrosis the most (Fig. 4c) as shown by the reduced number of CD68-positive macrophages as compared with human myoblast- and control-MPC-treated tissues 7 days post-CTX injury (Fig. 4d, e). Transplantation of human myoblasts, control MPC, and Givi-MPC in the muscle following 1 M post-CTX injury significantly decreased muscle necrosis compared to PBS-treated contralateral TA muscle (Figs. 5 and 6a–d). No significant difference in muscle fiber necrosis was observed between human myoblast- and control-MPC-treated TA muscle. However, Givi-MPC treatment reduced necrosis area compared to treatment with other MPC (Fig. 5e). Similarly, Givi-MPC transplantation reduced collagen deposits (red) compared to PBS-, human myoblast-, and control-MPC-treated muscle (Fig. 5f, g–j).

### Givi-MPC repopulated the muscle stem cell niches

A small number of Givi-MPC were transformed into muscle stem cells and occupied their sites under basal lamina as evidenced by double positivity for Pax7 and human lamin A/C at 1 M post-transplantation (Fig. 6a). A schematic outline of reinjury experiments with CTX is provided (Fig. 6b). Compared with the contralateral PBS-treated TA muscle, the expression of dystrophin was observed in the Givi-MPC-treated TA muscle after reinjury (Fig. 6c). Furthermore, the Givi-MPC-treated TA muscle showed increased muscle regeneration and reduced infiltration of inflammatory cells compared with the contralateral PBS-treated muscle (Fig. 6d). These data indicated that the engrafted Pax7-positive cells responded to reinjury and formed new muscle fibers. Muscle fiber fibrosis in TA muscle treated with Givi-MPC after reinjury was also decreased compared with contralateral PBS-treated muscle (Fig. 6e, f).

### Extracellular vesicles derived from Givi-MPC promoted angiogenesis in muscle following CTX injury

**Fig. 7:**
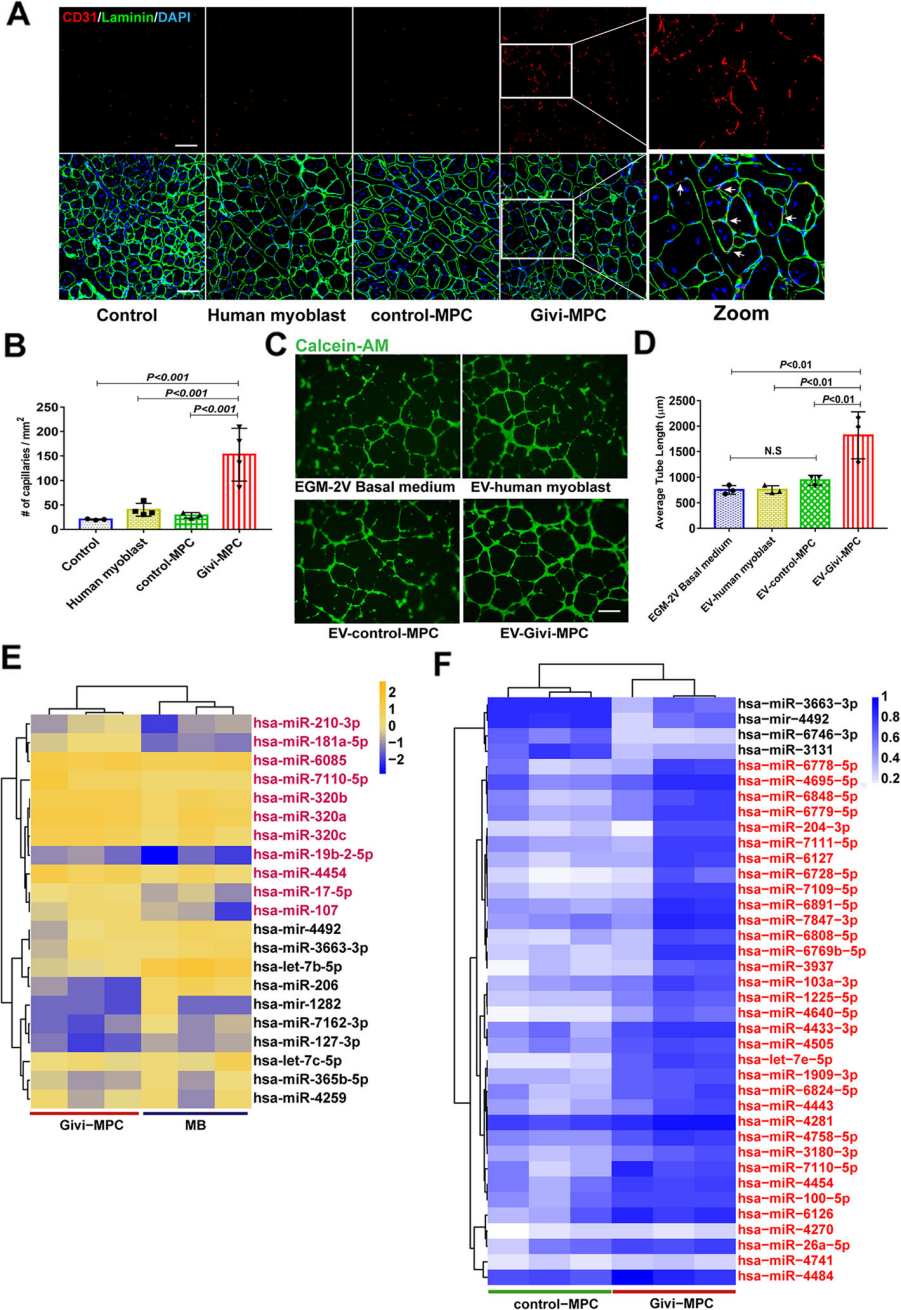
Extracellular vesicles derived from Givi-MPC promoted angiogenesis. **a** Representative images of CD31 (red) and laminin (green) staining in Mdx/SCID mice treated with MPC 1 M post-injury. Bar = 50 μm. The boxed area is shown at high magnification. **b** Quantification of capillary density (CD31-positive capillaries). **c** Representative images of tube formation by human aortic endothelial cells (HAECs) following EVs treatment from human myoblasts, or control-MPC or Givi-MPC (1 μg/well, 24-well plate). HAECs were labeled with Calcein AM (green). Bar = 500 μm. **d** Tube formation assay. Average tube length was analyzed from 3 biological repeated experiments. Heatmap showing upregulation of miRNAs in EVs derived from Givi-MPC compared to EV human myoblasts (**e**) and control-MPC (**f**)

EVs are small, membrane-bound vesicles released from cells that can transport cargo including DNA, mRNAs, microRNAs (miRNAs), and proteins [16, 18]. The secreted EVs carrying specific miRNA from Givi-MPC likely facilitated angiogenesis [16]. Angiogenesis is critical for muscle regeneration [19, 20]. Givi-MPC treatment resulted in higher capillary density (CD31 positivity) in the TA muscle 1 M post-CTX injury (Fig. 7a, b). Next, we tested whether increased angiogenesis was due to paracrine effects by EVs released from MPC. We isolated EVs from Givi-MPC using size exclusion columns. The size of the isolated EVs was approximately 118 ± 31.7 nm (Fig.S5A and B). The treatment with EVs from Givi-MPC promoted tube formation as shown by in vitro tube formation assay (Fig. 7c) with a higher average tube length (Fig. 7d) compared to the treatment with EVs from human myoblasts or control-MPC. We further analyzed the miRNA cargo contents of EVs from Givi-MPC. According to the heatmap analysis of the miRNA profiles in EVs, we showed differential expression of specific miRNAs within Givi-MPC EVs vs. human myoblast EVs (Fig. 7e, fold change > 1.5, and fold change > 4 in miRNAs enriched in EVs from Givi-MPC compared with EVs from control-MPC (Fig. 7f). Particularly, miR-210, miR-181a, miR-17, and miR-107 expression was elevated in EVs from Givi-MPC compared with EVs from human myoblasts. Let-7e-5p, miR-26a-5p, and miR-103a expression was increased in EVs from Givi-MPC compared with EVs from control-MPC.

## Discussion

In the present study, we successfully generated highly proliferative MPC from multiple hiPS cell lines using CHIR99021 and Givi. These cells expressed myogenic markers including Pax7 and desmin and were also capable to differentiate into muscle cells under specific differentiation medium in vitro. Of particular significance was the ability of these MPC to differentiate into muscle cells in the dystrophic mouse, making them more suitable for regenerative purposes. These cells possessed special properties which made them unique for therapeutic applications. Migration and engraftment of transplanted cells to the site of injury are crucial to initiate differentiation into skeletal muscle in the dystrophic muscle [21, 22]. Limited cell migration hampers engraftment efficiency in the skeletal muscle [23, 24]. In the present study, we found MPC induced by Givi exhibited superior migration and proliferation capabilities compared with human myoblasts and control MPC generated by CHIR99021 and FGF. GO analysis further confirmed the upregulation of cell migration-related genes enabling them to migrate to distant injured muscle [10]. In our data, genes related to cell migration were significantly upregulated by treatment with Givi-MPC. ITGA4 was the most upregulated gene with a 25.61-fold change. Integrin subunit α4 (ITGA4) is a member of the integrin alpha chain family of proteins. Integrin α subunits which pair with β1 play a critical role during in vivo myogenesis. Integrin α4 subunit is expressed in the myotome and in early limb muscle masses during muscle development [25, 26]. Murine Lbax1^+^ embryonic muscle progenitors also expressed ITGA4 [27]. It has been reported that teratoma-derived MPC exhibited robust engraftment in the muscle dystrophy model [10]. However, the mechanism of upregulation of ITGA4 by Givi-MPC and its role in migration needs further study. DMD is a disease with body-wide systemic and progressive skeletal muscle loss. Therefore, the role of ITGA4 in MPC migration would have profound importance in the MPC-based therapy for clinical application. In agreement with in vitro observations, we also observed robust engraftment of Givi-MPC compared to human myoblasts and control MPC upon transplantation in muscle tissue from Mdx/SCID mice following CTX injury. The novelty of these findings can be further appreciated by introducing gene-editing therapies for skeletal muscle diseases. CRISPR-Cas9-corrected DMD iPSC line could be differentiated into MPC for transplantation in order to replace dead muscle and restore dystrophin in the DMD muscle. The marked engraftment in the muscles of Mdx/SCID mice by human iPS-derived skeletal myogenic progenitors resulted in an increased number of dystrophin expressing myofibers or human laminin positive myofibers. However, it should be noticed that the therapeutic potential of Givi-MPC was investigated in a DMD mouse model with an acute muscle injury. Ideally, their therapeutic effects need to be investigated in the experimental conditions mimicking the chronic tendency of dystrophic muscle undergoing rupture upon usage. Besides dystrophin, the presence of neuromuscular junctions in human laminin positive myofibers together with dystrophin following Givi-MPC transplantation, suggest the formation of functional myofibers by transplanted cells.

Histological analysis showed that fewer muscle fibers had undergone necrosis and fibrosis in injured TA muscle of Mdx/SCID mice treated with Givi-MPC. Infiltration of inflammatory cells in general contributes to myofiber necrosis [28, 29]. Although Mdx/SCID mice are immunodeficient, it has been reported that M1 macrophages participated in skeletal muscle regeneration in SCID mice [30], suggesting a partial immune reactivity in these mice. It has been reported that Givi has potential anti-inflammatory effects [31, 32]. For example, Givi decreased inflammation in a mouse myocardial infarction model [32]. With HE staining, we found infiltration of a larger number of inflammatory cells in the TA muscle from Mdx/SCID mice treated with PBS, or human myoblasts or control MPC treatments 7 days post-CTX injury. On the other hand, a limited macrophage infiltration was observed in Givi-MPC transplanted Mdx/SCID mice 7 days post-CTX injury. Additionally, with TNFα stimulation, Givi-MPC suppressed the expression of IL6 compared with control-MPC. Similarly, the reduction of ROS generation by Givi-MPC after treatment with hydrogen peroxide further support our conclusion that Givi-MPC also possess antioxidative properties enabling them better survival and engraftment in the injured or dystrophic muscle. These observations support the notion that Givi-MPC had anti-inflammatory and regenerative effects upon transplantation in CTX-injured muscle. Given the anti-inflammatory properties of Givi, it is likely that molecular and biochemical properties of MPC reflect on the source of reprogramming molecule. Specific miRNA cargo of EVs from Givi-MPC is likely responsible for the anti-inflammatory effect. For example, miR-17 has been demonstrated to exhibit anti-inflammatory effects [33]. Besides immediate effects on engraftment and differentiation, the long-term maintenance of newly formed skeletal muscle is ultimately dependent on the ability of the transplanted MPC to contribute to the skeletal muscle stem cell pool [10]. Here, upon transplantation, we observed a few of Givi-MPC under the basal lamina which were positive for Pax7 and with subsequent reinjury these MPC contributed to the secondary regeneration in the Mdx/SCID mice. This observation supports our conclusion that a subpopulation of Givi-MPC can seed the stem cell pool important in injury repair.

Following CTX injury, an initial decline in skeletal muscle endothelial cells (EC) occurred in normal mice [34]. Although the number of EC can be restored near uninjured levels by day 14 post-injury in normal mice [34], the muscle vascular regeneration is likely impaired in Mdx mice. Angiogenic impairment of the vascular EC from Mdx mice has been reported [35] resulting in a marked decrease in the vasculature in the TA muscle of Mdx mice [36]. The local delivery of muscle-derived stem cells engineered to overexpress human VEGF into the gastrocnemius muscle of Mdx/SCID mice resulted in a marked increase in angiogenesis accompanied by enhanced muscle regeneration and decreased fibrosis compared with mice treated with non-engineered cells [37]. The ability of satellite cells isolated from Mdx mice was impaired to promote angiogenesis, as demonstrated in a co-culture model of satellite cells and microvascular fragments [38]. Here, the current study demonstrated that after Givi-MPC transplantation, an increase in capillary density was observed as evidenced by CD31 staining in CTX-injured Mdx/SCID mice compared to treatment with other MPC or PBS. These results enforce the idea that interaction between EC and MPC was important for myogenesis and angiogenesis both in in vitro and in vivo during skeletal muscle regeneration [19]. To further strengthen this observation, we found that EVs from Givi-MPC were enriched in several miRNAs related to myoangiogenesis including miR-181a, miR-17, miR-210 and miR-107, miR-19b, Let-7e-5p, miR-26a, and miR-103 compared with EVs from human myoblasts or control-MPC. Due to the role of EVs in cell-to-cell communication, these EVs-enriched miRNAs have been demonstrated to promote angiogenesis. Activation of miR-17-92 cluster promoted angiogenesis via PTEN signaling pathway, while EC miR-17-92 cluster knockout impaired angiogenesis [39]. miR-181a and miR-210 were also reported to promote angiogenesis [40–43]. miR-26a, let-7e-5p, and miR-103a have also been reported to regulate EC function and angiogenesis respectively while dysregulation of let-7e-5p impaired endothelial progenitor cell function [44]. miR-26a promoted angiogenesis of microvessel endothelial cells and prevented EC apoptosis [45]. Additionally, another EVs-miR-103 protected EC from apoptosis under oxidative stress [46]. Thus, it is very likely that Givi-MPC EVs partly interacted with resident EC to initiate angiogenesis in Mdx/SCID mice after CTX injury. However, the mechanistic effects by miRNAs in EVs from Givi-MPC need further investigation in the future.

## Conclusions

In summary, we successfully generated highly expandable MPC from multiple hiPS cell lines using CHIR99021 and givinostat. Givinostat-induced MPC were highly proliferative and migratory in nature, and their transplantation resulted in a marked and impressive myoangiogenesis and restored dystrophin in injured TA muscle compared to the treatment with control MPC or adult human myoblasts. More importantly, they also replenished the satellite cell compartment. It is concluded that hiPSCs reprogrammed into MPC by givinostat possessing anti-oxidative, anti-inflammatory, and muscle gene-promoting properties are an effective cellular source for the treatment of muscle injury and restoration of dystrophin in DMD muscle.

## Supplementary Information

**Additional file 1 **: **Figure S1** The schematic outline for inducing MPC.

**Additional file 2 **: **Figure S2** Measurement of reactive oxygen species and anti-inflammatory cytokines: (A) Representative images of fluorescence after treatment of control-MPC and Givi-MPC with 100 μM H_2_O_2_ and dihydrorhodamine 123 (DHR 123) for 24 h. (B) Quantification plot for DHR 123 fluorescence intensity. (C) Representative Western blot images of IL6 expression in control-MPC and Givi-MPC after treatment with 10 ng/ml TNFα for 24h. (D) Semi-quantitation of IL6 expression (*n* = 3).

**Additional file 3 **: **Figure S3** Western blot analysis of Givi-MPC shows induced acetylation of histones H3 (A) 7 days and 14 days at lysine 9 after differentiation compared with control-MPC at 7 days after differentiation. No significant changes were observed for acetylation of histone H4 (Lys8) in both control and Givi-MPC (B). *n* = 3.

**Additional file 4 **: **Figure S4** (A) Engrafted GFP positive Givi-MPC expressed dystrophin. Bar = 200 μm. (B) Dystrophin expression in Mdx/SCID mice after MPC transplantation at 1M after CTX injury and staining with non-human specific dystrophin. Bar = 100 μm. (B). Quantitation of engrafted fibers at 1M: human dystrophin positive fibers (*n* = 6).

**Additional file 5 **: **Figure S5** (A) Extracellular vesicles (EVs) isolated from Givi-MPC were visualized by transmission electron microscopy (TEM). (B) The size of isolated EVs from Givi-MPC was roughly 118 ± 31.7 nm.

## Data Availability

The raw data of the miRNA array is deposited in the GEO database (GSE155094). The datasets used and/or analyzed during the current study are available from the corresponding author on request.

## Abbreviations

DMD: Duchenne muscular dystrophy
hiPSCs: Human induced pluripotent stem cells
MPC: Muscle progenitor cells
EVs: Extracellular vesicles
CTX: Cardiotoxin
SCs: Satellite cells
EC: Endothelial cells
