# Supplementary figures and images for: Pluripotent stem cell-induced skeletal muscle progenitor cells with givinostat promote myoangiogenesis and restore dystrophin in injured Duchenne dystrophic muscle
Source: Stem Cell Res Ther. 2021 Feb 12;12:131. doi: 10.1186/s13287-021-02174-3 (PMC7881535; doi:10.1186/s13287-021-02174-3)

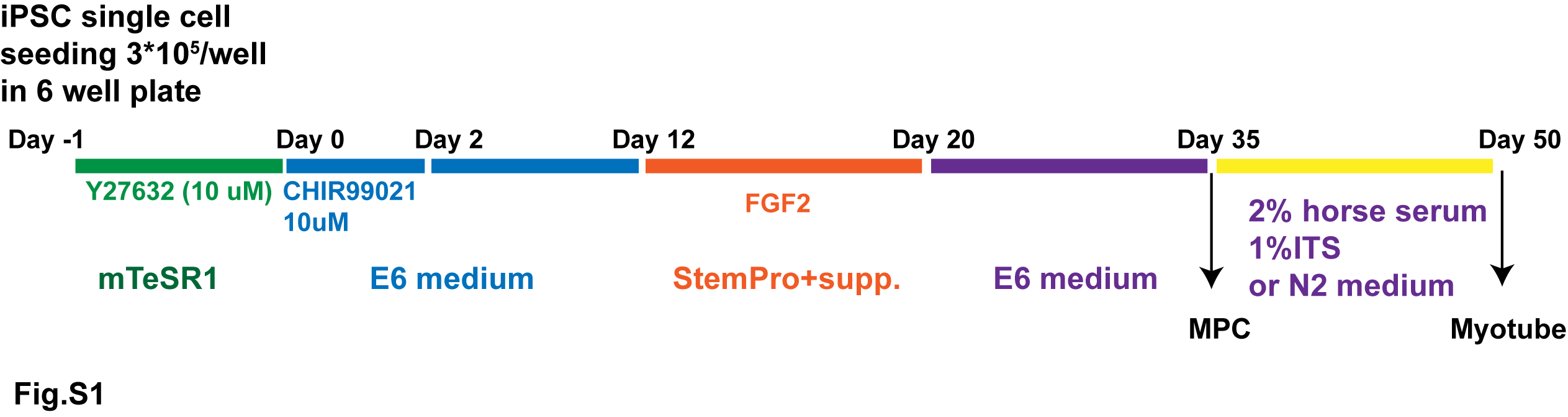

Supplement: Supplementary file 1 — Additional file 1 : Figure S1 The schematic outline for inducing MPC. [file 13287_2021_2174_MOESM1_ESM.tif]

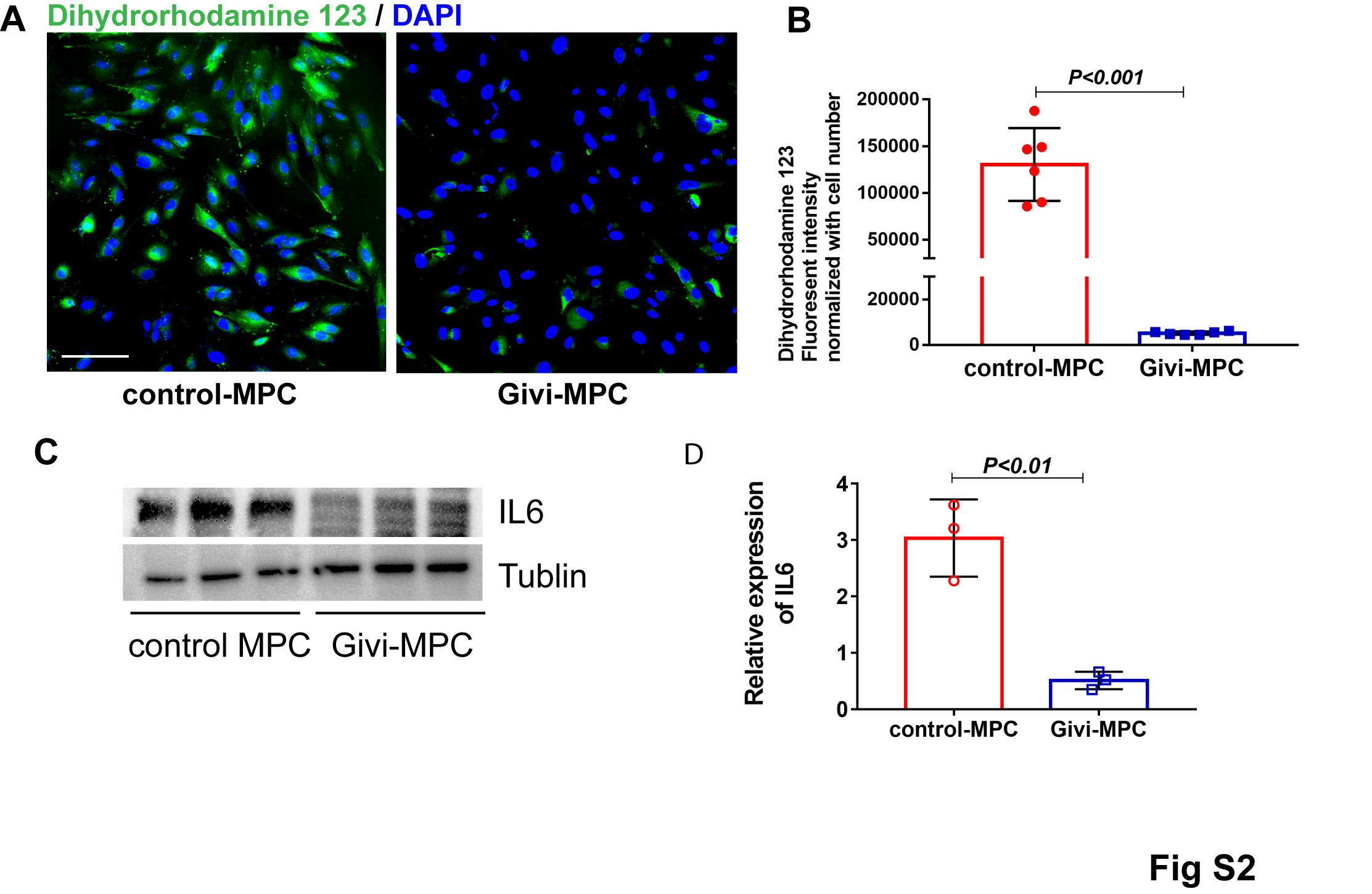

Supplement: Supplementary file 2 — Additional file 2 : Figure S2 Measurement of reactive oxygen species and anti-inflammatory cytokines: (A) Representative images of fluorescence after treatment of control-MPC and Givi-MPC with 100 μM H2O2 and dihydrorhodamine 123 (DHR 123) for 24 h. (B) Quantification plot for DHR 123 fluorescence intensity. (C) Representative Western blot images of IL6 expression in control-MPC and Givi-MPC after treatment with 10 ng/ml TNFα for 24h. (D) Semi-quantitation of IL6 expression (n = 3). [file 13287_2021_2174_MOESM2_ESM.tif]

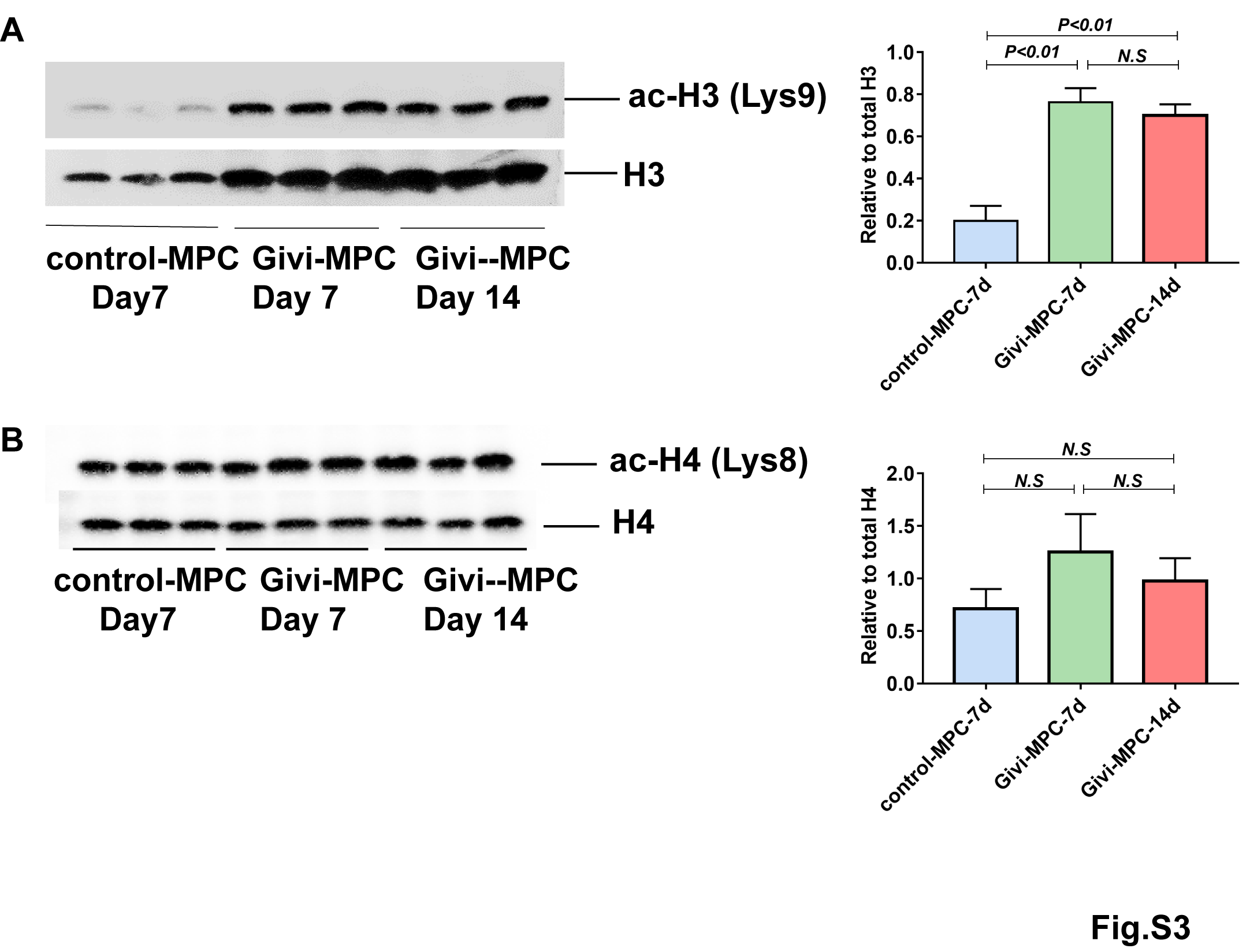

Supplement: Supplementary file 3 — Additional file 3 : Figure S3 Western blot analysis of Givi-MPC shows induced acetylation of histones H3 (A) 7 days and 14 days at lysine 9 after differentiation compared with control-MPC at 7 days after differentiation. No significant changes were observed for acetylation of histone H4 (Lys8) in both control and Givi-MPC (B). n = 3. [file 13287_2021_2174_MOESM3_ESM.tif]

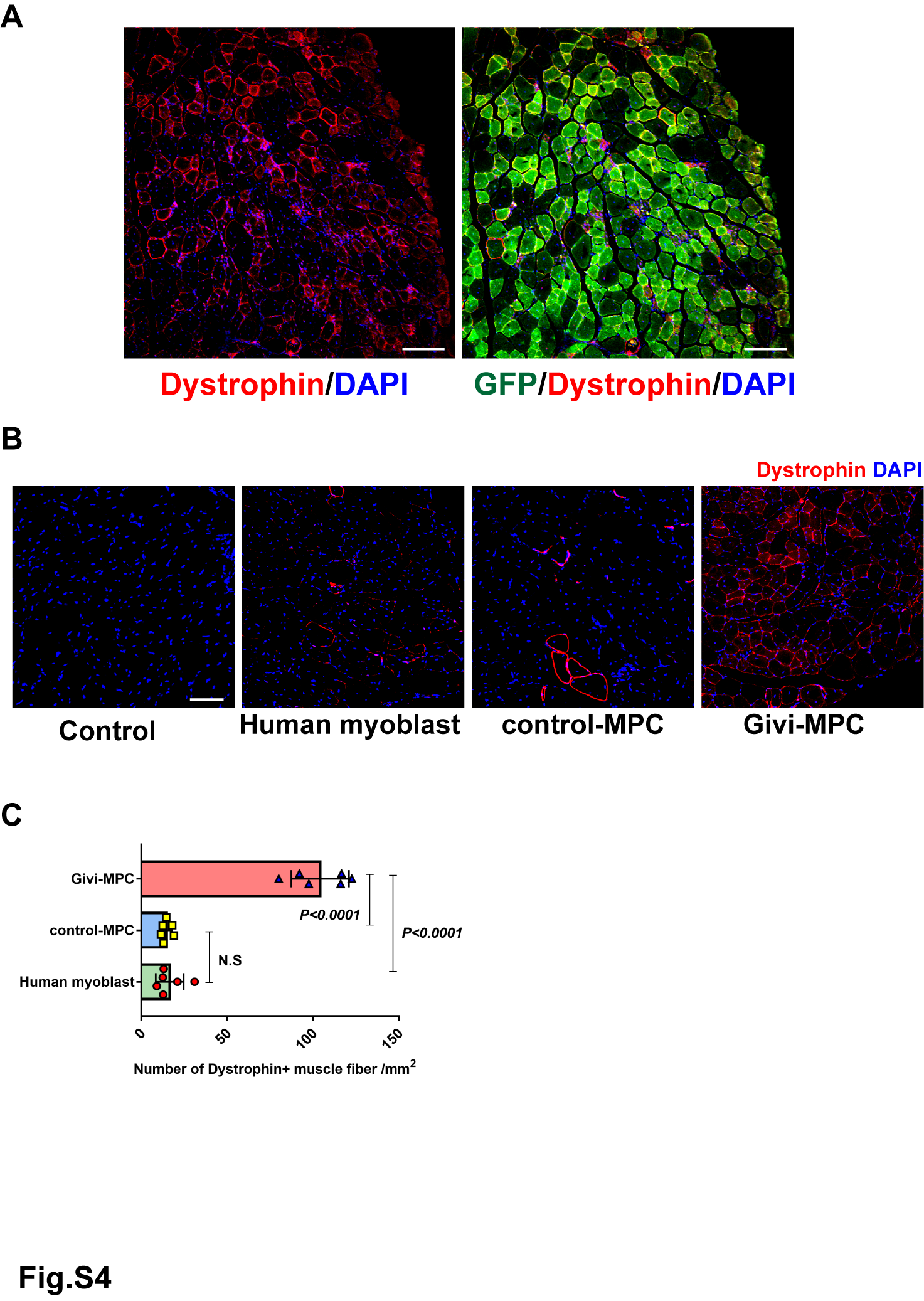

Supplement: Supplementary file 4 — Additional file 4 : Figure S4 (A) Engrafted GFP positive Givi-MPC expressed dystrophin. Bar = 200 μm. (B) Dystrophin expression in Mdx/SCID mice after MPC transplantation at 1M after CTX injury and staining with non-human specific dystrophin. Bar = 100 μm. (B). Quantitation of engrafted fibers at 1M: human dystrophin positive fibers (n = 6). [file 13287_2021_2174_MOESM4_ESM.tif]

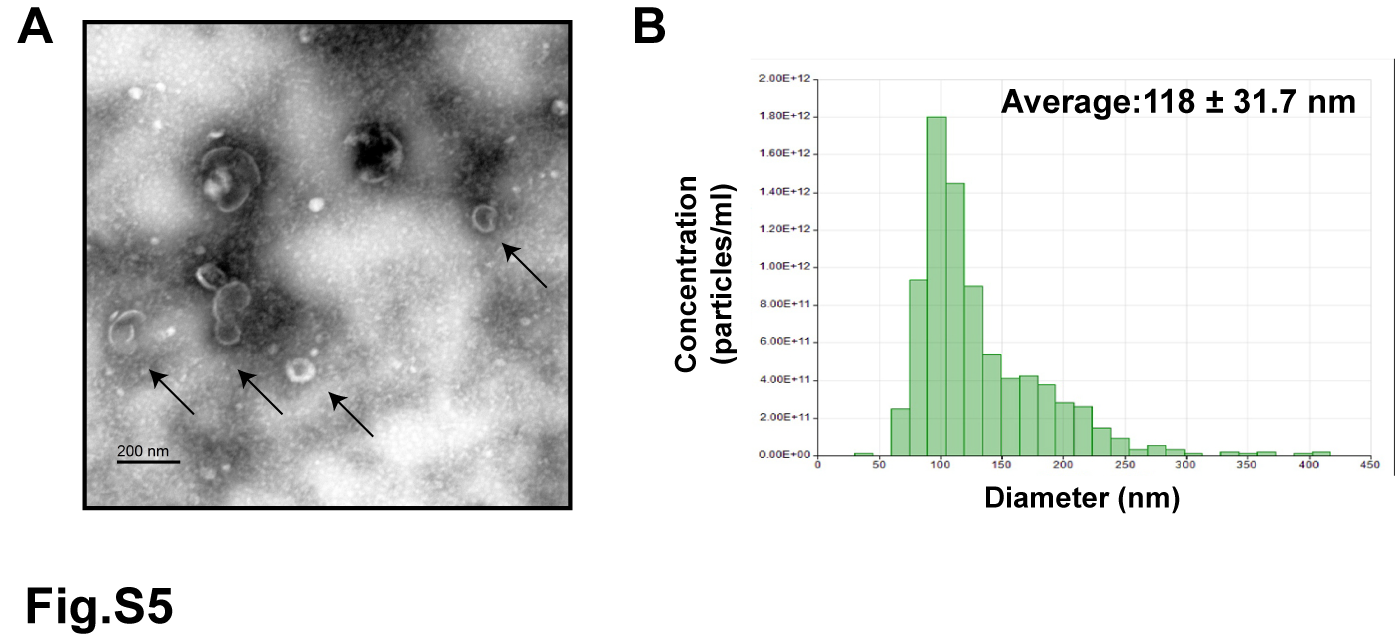

Supplement: Supplementary file 5 — Additional file 5 : Figure S5 (A) Extracellular vesicles (EVs) isolated from Givi-MPC were visualized by transmission electron microscopy (TEM). (B) The size of isolated EVs from Givi-MPC was roughly 118 ± 31.7 nm. [file 13287_2021_2174_MOESM5_ESM.tif]
